# Ion-Sensitive Field-Effect Transistor for Biological Sensing

**DOI:** 10.3390/s90907111

**Published:** 2009-09-07

**Authors:** Chang-Soo Lee, Sang Kyu Kim, Moonil Kim

**Affiliations:** 1 BioNanotechnology Research Center (BNRC), Korea Research Institute of Bioscience and Biotechnology (KRIBB), Daejeon 305-333, Korea; E-Mail: cslee@kribb.re.kr (C.-S.L.); tech81@hanmail.net (S.K.K.); 2 School of Engineering, University of Science and Technology (UST), Daejeon 305-333, Korea

**Keywords:** ISFET, ion-sensitive field-effect transistor, biosensor, biomolecules

## Abstract

In recent years there has been great progress in applying FET-type biosensors for highly sensitive biological detection. Among them, the ISFET (ion-sensitive field-effect transistor) is one of the most intriguing approaches in electrical biosensing technology. Here, we review some of the main advances in this field over the past few years, explore its application prospects, and discuss the main issues, approaches, and challenges, with the aim of stimulating a broader interest in developing ISFET-based biosensors and extending their applications for reliable and sensitive analysis of various biomolecules such as DNA, proteins, enzymes, and cells.

## 1. Introduction

For several decades, much attention has been paid to silicon-based biosensors in the field of bio-analytical applications due to their favorable characteristics, which include sensitivity, speed, miniaturization, and low cost. This interest is evident in the numerous studies that have monitored biological events such as nucleic acid hybridization s, protein-protein interaction s, antigen-antibody binding, and enzyme-substrate reactions using these silicon-based biosensors [1,2]. Among these, the ion-sensitive field-effect transistor (ISFET), is one of the most popular electrical biosensors, and has been introduced as the first miniaturized silicon-based chemical sensor. The ISFET, conventionally referred to as a pH sensor, has been used to measure ion s concentration s (H^+^ or OH^−^) in a solution, causing an interface potential on the gate insulator. The ISFET is a type of potentiometric device that operates in a way similar to the way the MOSFET (Metal Oxide Semiconductor Field-Effect Transistor) works. Therefore, in order to evaluate the performance of the ISFET, it makes sense to first understand the general principles behind the operation of the potentiometric sensor. The operating principle of the ISFET device is also covered in Section 2 of this review.

After the introduction of the ISFET biosensor by Bergveld in 1970 [3], and the first report by Caras and Janata regarding the use of an enzymatically modified ISFET for the direct detection of penicillin [4], numerous biosensors were established on the basis of theoretical development of ISFET technology. For example, there have recently been outstanding advances in the field of ISFET biosensors for use in biosensing research, including the progress of the enzyme-immobilized FET which detects H^+^ ion concentration, the DNA (deoxyribonucleic acid)-modified FET based on DNA hybridization detection, and the cell-based FET for cell metabolism sensing or the measurement of extracellular potential. Currently, the use of ISFET technology encompasses a wide range of applications in a variety of areas, and those in the biomedical and environmental monitoring areas are particularly noteworthy. In the following, this paper reviews recent advances and developments in the bio-analytical use of ISFET-based biosensors.

## 2. Operating Principle of FET-Based Biosensors

In general, a field-effect transistor (FET) consists of three terminals; the source, drain, and gate. The voltage between the source and drain of the FET regulates the current flow in the gate voltage. Specifically, the current-control mechanism is based on an electric field generated by the voltage applied to the gate. The current is also conducted by only one type of carrier (electrons or holes) depending on the type of FET (n-channel or p-channel). A positive voltage applied to the gate causes positive charges (free holes) to be repelled from the region of the substrate under the gate. These positive charges are pushed downward into the substrate, leaving behind a carrier-depletion region. The depletion region is populated by the bound negative charge associated with the acceptor atoms. These charges are “uncovered” because the neutralizing holes have been pushed downward into the substrate [5]. The positive gate voltage also pulls negative charges (electrons) from the substrate regions into the channel region. When sufficient electrons are induced under the gate, an induced thin n-channel is in effect created, electrically bridging the source and drain regions. The channel is formed by inverting the substrate surface from p-type to n-type (inversion layer). When a voltage is applied between the drain and source with the created channel, a current flows through this n-channel via the mobile electrons (n-type FET). In the case of a p-type semiconductor, applying a positive gate voltage depletes carriers and reduces the conductance, whereas applying a negative gate voltage leads to an accumulation of carriers and an increase in conductance (the opposite effect occurs in n-type semiconductors). The applied gate voltage generates an electric field which develops in the vertical direction. This field controls the amount of charge in the channel, and thus it determines the conductivity of the channel. The gate voltage applied to accumulate a sufficient number of electrons in the channel for a conducting channel is called the threshold voltage (V_TH_). Note that V_TH_ for an n-channel (p-channel) FET is positive (negative).

With these properties, the FET can be configured as a biosensor by modifying the gate terminal with molecular receptors or ion-selective membranes for the analyte of interest. The binding of a charged biomolecule results in depletion or accumulation of carriers caused by change of electric charges on the gate terminal. The dependence of the channel conductance on gate voltage makes FETs good candidates for electrical biosensors because the electric field generating from the binding of a charged biomolecule to the gate is analogous to applying a voltage to a gate. In general, the drain current of the FET-type biosensor is defined as follows:

(1) $$I_{\text{DS}}=1/2\mu \text{CW}/L{\left(V_{\text{GS}}-T_{\text{TH}}\right)}^{2}\;\;\;\;\;\text{at saturation region}\;(V_{\text{DS}}\geq V_{\text{GS}}-V_{\text{TH}})$$

(2) $$I_{\text{DS}}=\mu \text{CW}/L[(V_{\text{GS}}-T_{\text{TH}})V_{\text{DS}}-1/2{V^{2}}_{\text{DS}}]\;\text{at triode region}\;(V_{\text{DS}}<V_{\text{GS}}-V_{\text{TH}})$$

(3) $$C={\left[\sum \;\text{di}/(\varepsilon_{0}\varepsilon_{i})\right]}^{-1}$$

where μ is the electron mobility in the channel; W and L are the width and the length of channel, respectively; C is the net gate capacitance per unit area formed by the gate and the channel; di is the thickness of the dielectric; ε_0_ and ε_i_ is the dielectric constant of vacuum and deposited material, respectively.

**Figure 1.**
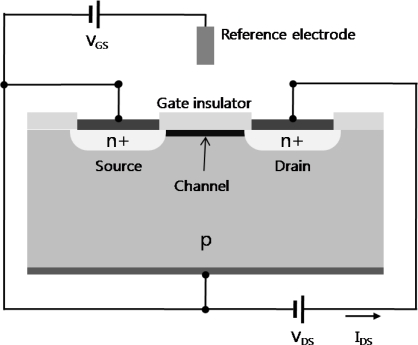
Structure of ISFET. It consists of source, drain, gate insulator, and reference electrode.

Generally, there are two types of planar FET-based biosensors, according to their structure; insulated-gate field-effect transistors (IGFET) and ISFET. In the case of IGFET, particularly MOSFET (metal-oxide-semiconductor field-effect transistor), the gate terminal is electrically isolated from the source and drain terminals. ISFET is similar to IGFET, but in the ISFET, the metal gate is replaced by an ion-selective membrane, electrolyte and a reference electrode (Figure 1). In the case of an ISFET biosensor, the amount of the current flow will be not only determined by the charges of biomolecules interacting on the gate dielectric, but also sensitive to pH, different ions, products of enzyme reactions, etc. An attractive feature of such FETs is that it is possible to detect biomolecular interactions in a label-free manner through a direct change in conductance or a related electrical property.

## 3. Applications of ISFET Biosensors

One distinct merit of the semiconductor-based biosensors like ISFETs, as opposed to optical systems, is their suitability for use in miniaturized measurement systems, thereby allowing its easy integration into the required electronics [6,7]. In this regard, an ISFET device of small size and low weight might be appropriate for use in a portable monitoring system, i.e., a hand-held drug monitoring system. When it comes to sensitivity and specificity of biosensor, both the fabrication of a nano-scale device and elimination of nonspecific molecular adsorption would contribute to an improvement in the limit-of-detection (LOD) and selectivity of the biosensor.

Investigators have conducted extensive studies in the electronic analysis of biomolecules by monitoring the variations in the charge density using ISFETs [8–22]. Currently, various kinds of biorecognition materials for biological analysis such as DNA, proteins, enzymes, and cells are being applied to ISFET measurements owing to the unique electrical and biological properties, thereby elevating the sensitivity and specificity of detection [4,23]. Among a variety of types of biosensors, one of the most promising approaches and the focus of investigators’ concerns is the ISFET-based biosensors and their integration in biological components. In the ISFET system based on different bio-contents for biological analysis, assorted concepts of biosensors like enzyme FETs, Immuno FETs, and DNA FETs that contain layers of immobilized enzymes, antibodies, and DNA strands respectively, have been reported in a large number of documents [23–28].

### 3.1. Applications of DNA-Based ISFET

When DNA strands bind to the gate surface of ISFETs, changes in surface potential occur due to the negative charge of DNA, thereby allowing for excellent performance of in DNA sensing. Through special treatments of the oxide layer of a FET, probe DNA can be immobilized onto the oxide surface in an orientation-controlled manner. As for methods of DNA detection, the most widely used techniques depend on enzymatic, fluorescent, and radiochemical tags. But all these methods, though they show high sensitivity and low detection limits, are insufficient to solve problems such as assay time, cost and complexity. To overcome these drawbacks, a label-free detection of DNA using a FET device with a real-time electrical readout system for rapid, cost-effective, and simple analysis of DNA samples has been proposed [8]. As an example of this, a detection platform based on an amorphous silicon-based (a-Si:H) ISFET for the label-free detection of covalent immobilization of DNA and subsequent hybridization of its complementary DNA was developed by Goncalves *et al.* [29]. In this study, DNA binding behavior was monitored using an ISFET biosensor, which was observed as changes in the threshold voltage (V_TH_). Through electric field monitoring, a sensitive response of a-Si:H ISFET to target DNA of different levels of hybridization was observed. Since the theoretical basis for elucidating the electronic data obtained from ISFET measurements is not strong, except for several parameters such as charge effect, capacitance effect, etc., a detailed study about the true behavior of thin-film FET biosensors will help to develop an advanced ISFET device suitable for real sample detection.

Detection of the hybridization of double stranded DNA was carried out using a single crystalline diamond synthesized by plasma-enhanced chemical vapor deposition (PECVD) by Nebel *et al.* [30]. To immobilize DNA onto the sensing layer of the constructed ISFET, amine linker-molecules were covalently bound onto hydrogen-treated diamond surfaces by photochemical method. Firstly, 3’-thiol-modified single stranded DNA was attached to the gate sensing layer of the diamond, and then the ssDNA-coated gate was hybridized with the complementary DNA. In the study, gate potential shift was determined to be between 30 mV and 100 mV with the reduced DNA surface density using a DNA-ISFET device. In general, the variation in surface conductivity can be explained from the transfer doping model, in which the increase in hole density will cause a decrease of pH value in the surface conductive layer of the diamond.

Estrela *et al*. employed MOS capacitors consisting of Au/SiO_2_/Si and Poly-Si TFTs with a gold metal gate as ISFET biosensor for label-free electrical detection of DNA hybridization [31]. When probe DNA bind to its complementary DNA, changes in electric potential in the electric double layer occur, leading to a shift in the C–V (capacitance–voltage) or I–V (current-voltage) characteristics. Their resuts showed that the discrimination of mismatched DNA was detected only 3 mismatched DNA, and no more. The authors proposed that by using this ISFET system with the appropriate DNA probes, it is potentially feasible to detect single-base-pair mismatches, revealing the possibility of the sensitive detection of single nucleotide polymorphisms (SNPs), one of the most frequent genetic alterations in the human population [32]. Since SNPs are commonly believed to be associated with response to drug and disease outcome, one of the most valuable applications of SNPs would be a biomedical application such as disease diagnosis and therapeutics. Along this line, Purushothaman *et al.* [33] suggested an application of ISFET technology for the detection of SNPs. In that study, the authors developed a useful procedure for sequencing one base via the detection of single-base mismatch in DNA.

**Figure 2.**
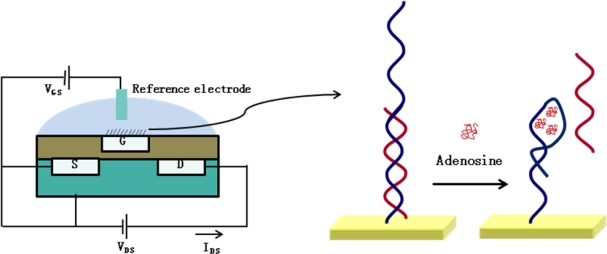
Label-free, reagentless aptasensor for adenosine using an ISFET device [34].

Regarding DNA biosensors, many studies on ISFET coupled with electronic aptamer-based (EBA) sensors have been reported [34]. Aptamers are nucleic acids (DNA or RNA [ribonucleic acid)] or peptide that selectively bind to their specific target molecules such as small molecules, nucleic acids, proteins, and even cells [35–37]. An ISFET-based aptamer sensor also uses a label-free electrochemical detection technology, measuring the changes in electrochemical signals generated from the interaction between the target molecules and the aptamers. In this context, Zayats *et al.* recently reported on the direct monitoring of adenosine, as a target molecule [34]. Upon the binding of adenosine to the cognate aptamer, changes in the electrical signal were monitored with an ISFET. Figure 2 shows the schematic diagram of an ISFET-based aptamer sensor for adenosine. Following a primary silanization of Al_2_O_3_ gate with 3-aminopropyltriethoxysilane, the surface was subsequently modified with glutaric dialdehyde. After the covalent immobilization of amine-functionalized aptamer on the gate surface, the hybridization of the nucleic acid to the aptamer was investigated by ISFET measurement. Consequently, the tested ISFET-based aptamer sensor exhibited the detection limit of approximately 5 × 10^−5^ M, and showed high specificity, as the aptamer-modified ISFET did not respond to other nucleotides, such as cytidine.

Besides the availability of ISFET-based DNA sensors, studies have raised the issue of the limitations of charge detection of DNA hybridization based on FET-type biosensors. In particular, the relatively thick insulator on top of the gate oxide can conspire to inhibit the effective detection of charges, because the thickness of the insulator may have a negative influence on the conversion of the charged DNA from the electrolyte to the sensing layer. To get around this, Song *et al.* [38,39] proposed a diamond solution-gate FETs (SGFETs), in which the surface is not covered by thick insulating layers, and DNA is immobilized directly onto amine-terminated sites, which is a critical factor in terms of sensitivity compared to existing DNA ISFET sensors. The diamond surface channel attached by DNA was exposed directly to the electrolyte lacking gate insulator, thereby leading to a large potential window for diamond up to >3.0 V. The tested device could rapidly detect 3-mer mismatched DNA, and potentially showed the possibility for the monitoring of single-base mismatched DNA, without sacrificing sensitivity. In addition, when DNA hybridization reaction takes place at the distance beyond the Debye length from the sensing layer, FET device fails to DNA detection. Thus, DNA hybridization measurement should meet the prerequisite where an electric field caused by redistributed charged molecules exists within the Debye length of the given solution.

### 3.2. Applications ISFET for Electro-Immunological Sensing

The pH sensitive ISFET is the most popular device as an immunosensor with a large range of insulators (SiO_2_, Si_3_N_4_, Al_2_O_3_ and Ta_2_O_5_) [40]. Regarding bio-recognition elements, antibodies are the most commonly used capture agents, enabling the identification and quantification of individual analytes, due to the specificity of the antigen-antibody interaction. An immuno-ISFET is composed of an antibody recognizing antigen coated onto the gate, and can be applicable for clinical diagnosis, thereby being increasingly emphasized [41]. When it comes to medical application of the immune-ISFET biosensor, the concept regarding “Debye screening length” should be taken into consideration. As well-characterized, the electric field fades away beyond the “Debye screening length”, which is the distance where moving charge carriers screen out the external electric field. Therefore, this Debye screening length has become one of the main disadvantages in measuring the biomolecular recongition using an FET-type biosensor. For this reason, it is necessary for FET measurements that biological sensing should take place within the Debye length (λ_D_). Considering this, it is noteworthy that diagnostic monitoring might be restricted with immune-ISFET because biomolecular interaction events in immuno-ISFET usually occur beyond approximately 10 nm from the gate surface due to the height of antibody. Accordingly, with the drawback of immuno-ISFET in the medical diagnosis application, only a few examples of ISFET-based immunosensors could be found in the literature [42–45].

**Figure 3.**
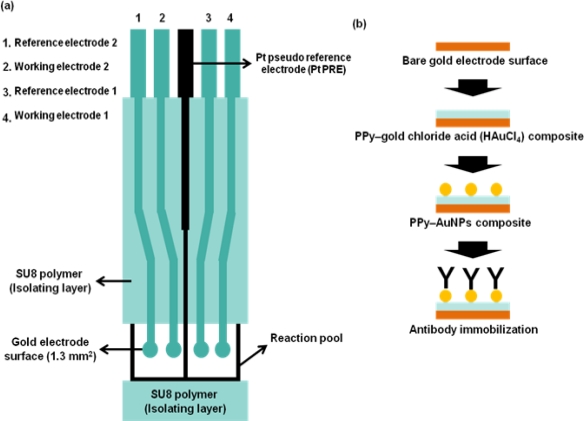
Schematic diagrams of the micro-potentiometric immunosensore (a), and antibody immobilization strategy (b) [48].

As an example of immuno-ISFET, Starodub *et al*. [43] developed an immunosensor based on ISFET for the determination of simazine, a s-triazine-type of herbicide. Following their research, two types of methods were employed for simazine detection - one is competitive immune analysis, and the other is sequential saturation of antibodies. Upon antibody binding to the simazine, the catalytic activities of peroxidase conjugated to antibody were determined, shown as a sign of pH shift after treatment with H_2_O_2_. Meanwhile, a great number of researchers have reported about evaluating protein adsorption to the FET surface, and modulating the static ISFET response [25,46], which depends on the discrete charge that proteins carry. There was a report on an alternative potentiometric method about detecting protein on the gate of ISFET [47]. The surface of the ISFET is covered with a monolayer of ligand-bound amino beads (0.9 μm diameter) which provide specific binding properties. This approach provided a generic method for coating the ISFET with a solid phase for immunochemical reaction. Typically, potentiometric immunosensors need the immobilization of immunoactive biomolecules such as antibody. As an alternative method for ISFET surface modification, the use of conducting polymers has been considered due to their high chemical stability, biocompatibility, and facility to be doped. Qu *et al.* showed a simple and direct process for the formation of composite film of conducting polymer-Au nanoparticles with the electrochemical growth of Au nanoparticle in conducting polymer, polypyrrole (PPy) [48]. Based on this method, an ISFET-type micro-potentiometric Hb/HbA1c immunosensor was developed and hemoglobin (Hb) and hemoglobin-A1c (HbA1c) in whole blood were detected with a highly improved sensitivity and immobilization of antibody onto gold electrode and the sensitivity. Figure 3 shows the pattern of the electrode and antibody immobilization based on electropolymerized polypyrrole–gold nanoparticles composite.

**Figure 4.**
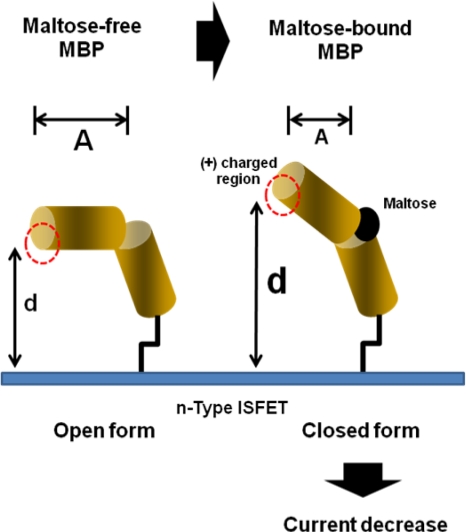
Schematic diagram of the ISFET-based monitoring via conformational changes in MBP [49]. Upon binding to maltose, MBP undergoes a structural transition into closed conformations, resulting in decrease of drain current.

Considering that miniaturization is a general trend in biosensor development, the proposed miniaturized electrode fabricated by MEMS technology is believed to be equal to this tendency. In the future, the electrode chip may be integrated with an ISFET device, hinting at the potential for it to be applicable for the development of system-on-chip (SoC) devices. More recently, an attractive approach for exploring conformationally changed protein has been reported by Park *et al.* [49]. In that study, a new type of ISFET charge sensor was developed for the determination of maltose-induced conformational change in maltose binding protein (MBP). As shown in Figure 4, when treated with maltose on the MBP-modified ISFET surface, the structural transition of MBP took place, leading to a substantial drop in the drain current of the ISFET device. The authors demonstrated that the ISFET charge sensor can be effectively utilized to evaluate the intramolecular conformational change in proteins.

### 3.3. Applications of Enzyme Based-ISFET

In general, enzyme FETs are based on the principle of pH-sensitive ISFETs in which the concentration of hydrogen ions during an enzymatic reaction is proportional to the level of substrate. So far, a variety of enzyme FETs have been developed for the detection of numerous analytes such as glucose, penicillin, urea, pesticides, phenolic compounds, steroidal glycoalkaloids, creatinine, etc [50–67]. During the last few years, rigorous efforts have been made to improve enzyme FETs for the purpose of resolving several issues such as stability, reproducibility, and compatibility of the FET system. Although the possibility of ISFETs as enzyme sensors was first suggested by Janata and Moss in 1976 [23], a practical application of ISFET as a penicillin-responsive device was first reported in 1980 [4]. To detect penicillin, they exploited a concept of the ISFET-based enzyme biosensor that employs two pH-sensitive ISFETs, one of which possesses a membrane containing cross-linked albumin-penicillinase, and the other which has a membrane of only cross-linked albumin membrane. Through their study, the authors evidenced that the tested enzyme-based ISFET might be suitable for the rapid detection of small amount of analyte with high sensitivity. Also, since it can provide the feasibility to save the analysis time through the automated penicillin measurement system based on ISFET, this ISFET-based enzyme biosensor has been suggested for the analysis of complex samples.

Another approach of ISFET for the monitoring of target proteins is the use of proton or hydroxyl ion produced during protein hydrolysis in response to trypsin. For example, Marrakchi *et al*. developed the ISFET-type trypsin biosensor for the determination of the substrate (BAEE, α-benzoyl-l-arginine ethyl ester hydrochloride) [68], thereby potentially showing the feasibility of the ISFET application to monitor small pentapeptides composed of five amino acids. The authors proposed the applicability of the developed biosensor to use for quality control in the cosmetic industry, because the tested small peptide is currently used in cosmetics formulation as peptide ingredients. Along this line, it is also possible to examine the protein concentration from the product of protein digestion using pH-sensitive electrodes. As an additional example of enzyme-based ISFETs, an electrochemical bi-enzyme sensor for the quantitative protein determination has been proposed [69,70]. The corresponding biosensors are based on the bi-enzyme system containing two enzymes, protease and oxidase. Recently, Freeman *et al.* developed a chemically modified-ISFET fabricated by incorporating 3-aminophenylboronic acid for analyzing the behaviours of dopamine and tyrosinase (TR) [71]. Upon formation of the boronate–dopamine complex on the gate surface, variations in the electrical potential were clearly observed, and then the detection limit of 7 × 10^−5^ M was determined, showing good performance of this system for the monitoring of dopamine and TR.

So far, a variety of the detection methods for TR activity monitoring such as the use of metallic nanoparticles [72,73], semiconductor QDs [74], and functionalized redox molecules [75] as optically or electrochemically active labels have been developed. The method developed by Freeman *et al*. has demonstrated a label-free procedure for measuring TR activity with comparable sensitivity to previous methods [71]. A novel potentiometric biosensor allowing quantitative analysis of the proteinases through their esterase activity has been established by Biloivan *et al.* [76]. The device was created using a pH-FET and an immobilized α_2_-macroglobulin-trypsin complex. This pH-sensitive ISFET biosensor showed a linear correlation with esterase activity in the range from 0.1 to 30 U/mL, and exhibited good stability and reproducibility. The authors proposed that the same detection procedure for trypsin using pH-sensitive FET system could be also applicable to the simultaneous detection of other trypsin-like proteinases. As for ISFETs for toxicity monitoring, potentiometric and conductometric biosensors coupled with cholinesterases for the detection of the toxic substances in the environmental control have been developed [77–81]. These biosensors based on enzyme inhibition mechanism have proved to be suitable for the early warning detection system to determine the presence of dangerous chemicals. A variety of toxins including organophosphorous pesticides and carbamate pesticide were analyzed via the cholinesterase-based biosensor.

Additionally, some material biosensors with photopolymers and various polymers for enzymatic biosensors have been developed by many researchers [57,82–85]. Recently, Rebriiev *et al.* [59] described an ISFET based enzymatic biosensor for urea measurement, commonly referred to as ISFET urea sensor. In that work, a simple and rapid enzyme immobilization method on the ISFET gate surface was adopted on the basis of LPhPC (liquid photopolymerizable composition), in which the resultant polymer could be formed under UV. The developed ISFET urea sensor showed a considerable improvement in sensitivity, detection limit, and response time, as the linear response is in the range of 0.05–20 mM, and response time 5–10 min, suggesting that the developed ISFET urea sensor has potential for clinical application for the analysis of urea in blood samples.

**Figure 5.**
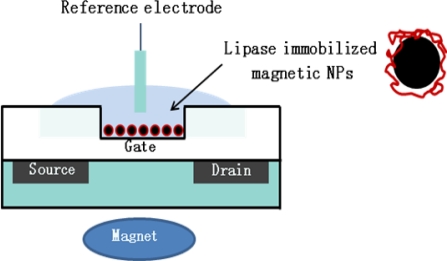
Schematic representation of the ISFET braced to a permanent magnet [87].

Regarding target immobilization, for the purpose of the stable immobilization of enzymes to the ISFETs gate surface, a variety of methods have been developed including covalent attachment or polymer entrapment [54,86]. For example, Vijayalakshmi *et al*. [87] developed a method for maintaining the target enzyme at the gate sensing using magnetic nanoparticles, where lipase was immobilized onto magnetic nickelferrite nanoparticles, and then the resulting enzyme-modified nanoparticles could be retained at the gate surface because the magnet continued to run from the bottom of ISFET gate, as shown in Figure 5. The advantages of the proposed method underlie (1) improved mass transfer, (2) possibility to be applicable for use in a multiple detection system based on FET device, and (3) increased amount of bound enzyme on the surface.

Recently, attention to adenosine triphosphate (ATP) sensing has been growing because ATP is the main energy carrier in all living organisms, and thus energy cannot be produced in the body without ATP. Thus, an enzyme- functionalized ISFET for ATP sensing was developed by Migita *et al.* [88]. The enzyme immobilized on a Ta_2_O_5_–modified-ISFET surface catalyzes the dephosphorylation of ATP, which is followed by the accumulation of protons at the gate because the enzymatic reaction produces H^+^ as a by-product. The authors demonstrated that the ISFET response was highly proportional to the concentration of ATP in the given solution, suggesting that this ISFET ATP sensor might be effective enough to provide a real hygienic measurement.

Although ISFET-based enzyme sensors offer excellent activity and reliability, it could be restricted on buffer conditions such as pH or buffer capacity, because the ISFETs exploit the operation theory that measure the pH changes caused by an enzyme-catalyzed reaction at the gate surface, which is highly influenced by the buffer conditions [1,89]. To overcome this problem, several approaches such as the use of the additional charged polymeric membranes, and buffer solutions with low capacities have been attempted by Volotovsky *et al*. [90]. As another example of enzyme FET, Ishige *et al.* [91] developed an extended-gate FET-based enzyme sensor that enables the measurement of the changes in the redox potential derived from enzyme-catalyzed reaction. Although the sensitivity of ISFET-based enzyme sensors is strongly affected by buffer conditions, the tested sensor was not influenced by pH change or buffer capacity. From their results, it is likely that the enzyme-catalyzed reaction via the chemical reaction is responsible for the potential changes rather than pH variation under the developed extended-gate FET sensor. More recently, in order to improve the performance of general ISFETs, another type of ISFET, the so-called region ISFET (RISFET), has been proposed by Risveden *et al*. [92]. The developed RISFET has an extraordinary feature in that its performance depends on the electrical field strength generated by ion molecule reaction occurring in a unique region flanked by the sensing electrodes. The authors proposed that the RISFET device could be a promising nanobiosensor, because it allows for the dielectrophoretic trapping of a single enzyme, thereby allowing the analysis of small volumes of fluid containing small amount of analytes.

### 3.4. Applications of ISFET for Monitoring Living Cell Responses

Cell-based biosensors, which provide a variety of biologically active information for analytes allow for the monitoring of cytotoxic effects in response to dangerous substances, as well as the physiological cell response to numerous stimuli. Therefore, living cells are utilized as their bio-contents, and the activity of living cells can be electrochemically monitored in a cell-based biosensor system. With these benefits, cell-based sensor systems have been considered as a promising technology for biomedical and pharmacological applications [93]. In particular, increasing attention has been paid to the integration of live cells with silicon-based FET devices. Above all, cell-based ISFET biosensors are of great interest for upcoming applications in the area of neuronal network and transmission paths of ionic channels.

**Figure 6.**
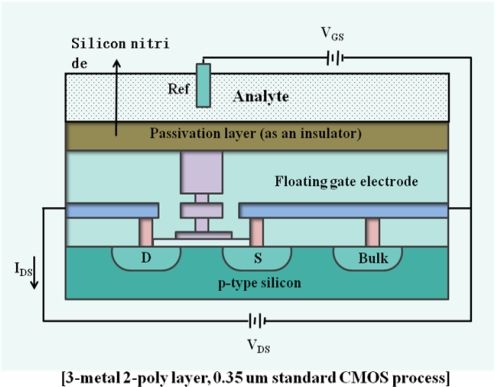
A cross-sectional diagram of an ISFET using an unmodified CMOS process [95].

When it comes to cell-based ISFET applications, some cellular phenomena such as cellular respiration and acidification have been monitored simultaneously. Lehmann *et al*. reported pH-dependency in changes of the extracellular acidification and respiration rates under the same cell culture fluid [94]. Recently, Milgrewa *et al.* reported the fabrication of the sensor array chip based on pH-sensitive ISFET for the direct extracellular imaging [95], which is composed of a 16 pixel by 16 pixel array of ISFETs that have biopotential readout circuits for signal acquisition systems. As shown in Figure 6, each pixel array has a floating gate structure which is passivated by a pH sensitive membrane.

Each ISFET operates as a pH sensor with a linear operating range of 2.5 V, threshold voltage of −1.5 V and a sensitivity of 46 mV/pH. With the performance of the image sensor, the topography of the chip could be analyzed with the established MDCK (Madin Darby Canine Kidney) cell line by measuring the pH changes.

**Figure 7.**
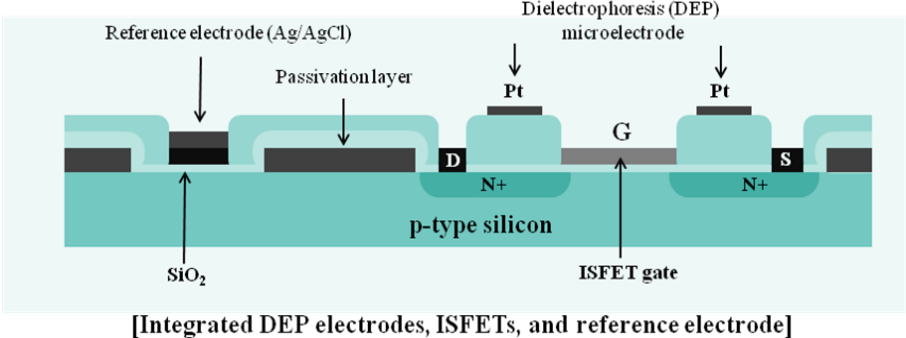
A cross-sectional diagram of the device showing the ISFET sensors, DEP microelectrodes, and pseudo-reference electrode [98].

Meanwhile, development of a cell-based biosensor platform using FET array was achieved for extracellular signal recording, commonly used in pharmacological tools, in a way non-destructive manner [96]. Also, monitoring of extracellular ion concentration-dependent changes in membrane potential in response to a variety of chemical stimuli has been developed on the basis of a cell-based biosensor. For example, Wang *et al.* established a non-invasive monitoring system for studying ion channels modulation, which is the most evaluated potential target for therapeutics [97]. They designed an ISFET sensor array to detect the concentration of extracellular ions, such as Na^+^, K^+^, and Ca^2+^, showing that their ISFET biosensors allowed for non-destructive, real-time and long-time analysis of cell coupling. For the use of cells in a biosensor device, it is essential to localize the cells in the active area of the sensor, which can be achieved by dielectrophoresis (DEP). As shown in Figure 7, the sensor consists of integrated DEP electrodes for cell positioning, ISFETs, and reference electrode [98]. Using the developed ISFET device with integrated DEP electrodes as a tool for characterizing bacterial positioning, bacterial metabolism was also detected over a few hours by monitoring pH variation after adding glucose. When glucose was added, uptake and consumption of glucose took place by the bacterial cells. When the oxygen supply was limited, the bacterial cells metabolized glucose thereby dropping the pH. Also Castellarnau *et al.* showed that the typical performance of pump stop and flow could be achieved by using one ISFET with cells (LS 174T adenocarcinoma colorectal cell line) and the other ISFET without cells [99]. Their cell-based ISFET system is expected to be practically used for bacteriological applications, for example, monitoring of sugar consumption metabolized by various types of bacteria.

The silicon-based sensor/chip system for biological applications has become a good alternative in detection technology, which is based on analyzing parameters of cell viability. For example, ISFETs and planar electrodes were optimized to examine *in vitro* or *in vivo* cell functions such as cellular metabolism [94,100–102], attachment and spreading of epithelial MDCK cells using electric cell-substrate impedance sensing (ECIS) [103], and cell response to different toxic materials [104–107]. Recently, on-line and continuous system based on ECIS for the monitoring of cell growth and cytotoxic effects of metal compounds on fibroblastic V79 cells has been developed by Xiao and Luong [105]. Likewise, Ceriotti *et al.* have also established on-line monitoring system for exploring cellular metabolism, adhesion, and the cytotoxicity in response to metal ions in immortalized mouse fibroblasts (BALB/3T3) using a multiparametric chip-based system [108], which can evaluate the extracellular acidification rates (ECAR) with pH-sensitive ISFETs in a parallel and label-free manner. For another example for a cell-based ISFET, on-line monitoring of cellular functions such as respiration and acidification has been took place by utilizing a single complementary CMOS ISFET [94]. That kind of cell-based CMOS ISFET was capable of providing knowledge on single cell dynamics, for example, chemical dynamics in cell metabolism such as aging inhibition, apoptosis regulation, and glycolysis inhibition. Consequently, a cell-based ISFET biosensor would offer a high fidelity system for on-line monitoring of the pharmacodynamic effects of cell behavior in response to a variety of chemical stimuli.

## 4. Conclusions

Since the ISFET was invented, ISFET biosensors have provided various opportunities for developing a new generation of biosensor technologies. Because of their simple and clear operation principles, ISFET biosensors have a well-established position as a powerful sensing tool for detecting DNA, proteins, enzymes and cells. Although various types of ISFET-based biosensors have been developed, they still suffer from a variety of fundamental and technological problems such as impurities of the semiconductor and instability of functional groups in the sensing layer. To overcome these problems, interdisciplinary cooperation from various research fields such as chemistry, biology, electrics, and physics must be required.

In the future, ISFET biosensors may have advanced performance and special properties, accompanying nanomaterials such as nanoparticles, nanotubes, and nanowires. The integration of ISFET in microsystems such as micro-total-analysis-system (μ-TAS) or lab-on-a-chip (LoC) may also provide small packaged sensor systems with ultrahigh sensitivity. We believe that these ISFET biosensors can be useful for biomedicine, clinical diagnosis, environmental monitoring, and point-of-care-testing (POCT) system.
